# Intercellular Communication in the Central Nervous System as Deduced by Chemical Neuroanatomy and Quantitative Analysis of Images: Impact on Neuropharmacology

**DOI:** 10.3390/ijms23105805

**Published:** 2022-05-22

**Authors:** Diego Guidolin, Cinzia Tortorella, Manuela Marcoli, Guido Maura, Luigi F. Agnati

**Affiliations:** 1 Department of Neuroscience, Section of Anatomy, University of Padova, 35121 Padova, Italy; cinzia.tortorella@unipd.it; 2 Department of Pharmacy, Center of Excellence for Biomedical Research, University of Genova, 16126 Genova, Italy; marcoli@difar.unige.it (M.M.); maura@difar.unige.it (G.M.); 3 Department of Biomedical Sciences, University of Modena and Reggio Emilia, 41125 Modena, Italy; luigi.agnati@gmail.com

**Keywords:** volume transmission, cotransmitters, receptor–receptor interactions, cellular networks, histochemistry, image analysis

## Abstract

In the last decades, new evidence on brain structure and function has been acquired by morphological investigations based on synergic interactions between biochemical anatomy approaches, new techniques in microscopy and brain imaging, and quantitative analysis of the obtained images. This effort produced an expanded view on brain architecture, illustrating the central nervous system as a huge network of cells and regions in which intercellular communication processes, involving not only neurons but also other cell populations, virtually determine all aspects of the integrative function performed by the system. The main features of these processes are described. They include the two basic modes of intercellular communication identified (i.e., wiring and volume transmission) and mechanisms modulating the intercellular signaling, such as cotransmission and allosteric receptor–receptor interactions. These features may also open new possibilities for the development of novel pharmacological approaches to address central nervous system diseases. This aspect, with a potential major impact on molecular medicine, will be also briefly discussed.

## 1. Introduction

Since the work of Golgi (1843–1925) and Cajal (1852–1934), microscopy has been one of the neuroscientist’s cardinal tools. When used together with Golgi’s technique for staining a sparse population of cells (see [1]), the morphological approach provides the data that drove the famous debate between Golgi [2] and Cajal [3] about whether the nervous system was composed of single cells or a syncytium, leading Sherrington [4] to introduce the concept of ‘synapse’ to describe a region of discontinuity between neurons where communication between them could occur. Thus, morphological features of the nervous cells, their localization, and their possible network organization became a significant line of research to investigate the integrative functions of the brain. 

In the last decades, indeed, a large body of new evidence on brain structure and function has been acquired by morphological investigations based on synergic interactions between chemical neuroanatomy approaches (such as histology, histochemistry, and immunocytochemistry) [5] and new techniques in microscopy (as, for instance, confocal microscopy or atomic force microscopy), as well as in brain imaging. A next key step was the quantitative analysis of the obtained images, mainly achieved by the application of computer-assisted image analysis methods [6]. This complex combination of different approaches has produced a deeper view on brain structure and function, illustrating the central nervous system (CNS) as a huge network of cells, regions, and systems in which intercellular communication processes virtually determine all aspects of the integrative function performed. In other words, investigations on the brain integrative functions should consider “complex cellular networks”, that is, functional networks including not only neurons but also the other cell populations in the CNS [7].

The key role played by the network architecture as a structural substrate for the CNS functions also represents the main rationale for the emerging field of connectomics, the comprehensive study of all aspects of CNS connectivity [8,9]. Moreover, the new quantitative morphological approaches allowed for the characterization of brain structure and function in both physiological and pathological conditions, leading not only to the emergence of new “paradigms” [10] concerning intercellular communication in the CNS, but also to the development of new therapeutic approaches with a possible significant impact on neuropharmacology.

These aspects, in which our group was deeply involved, will be the main focus of the present review article, since they may indicate possible stimulating new challenges for the study of CNS dynamics and for the development of new treatment strategies for CNS diseases.

## 2. Intercellular Communication in the CNS

About 30 years ago, the classical view on the intercellular communication in the CNS was broadened by the proposal of a classification involving two main modes of communication, called “wiring” (WT) and “volume” (VT) transmission [11]. The main criteria that allow for the differentiation of WT from VT are the characteristics of the communication channel and, more precisely, the physical boundaries of the channel. 

WT, indeed, is characterized by a well-identified physical channel (i.e., “a wire”) through which the cell source of the signal is connected to the target cell, while VT is characterized by the three-dimensional diffusion of signals (as Golgi originally proposed on the basis of Volta’s studies on the second class of electrical conductors), such as transmitters, trophic factors, gases, and ions, which reach the CNS and/or can be released in the extracellular space and in the cerebrospinal fluid by any type of cell (see [9] for a recent review). This proposal was influenced by previous important contributions on communication in the CNS [12,13,14,15] and was mainly based on a number of observations obtained by the combination of chemical anatomy techniques with quantitative analytical approaches. These aspects will be briefly examined in the sections that follow.

### 2.1. Wiring Transmission

The most important example of WT is classical synaptic wiring. It is well known that communication between neurons occurs through specific channels, the synapses, and involves endogenous chemical messengers collectively called neurotransmitters. A compound is referred to as a neurotransmitter when it meets four criteria. First, it is synthesized in the neurons; second, it is present in the presynaptic terminal and released in sufficient amounts enough for exerting a certain action on the postsynaptic neuron by targeting specific receptors; third, the exogenous administration has to mimic the endogenously produced neurotransmitter’s action; and last, there are intrinsic mechanisms responsible for its removal from the site of action [16]. We presently know about 200 substances acting as neurotransmitters in the human CNS that have been classified on the basis of their chemical, functional, and molecular properties along with their location in the CNS [17]. In this respect, a methodological breakthrough in the chemical neuroanatomy field was the introduction of the formaldehyde monoamine fluorescence technique by Falck and Hillarp in 1962, allowing the conversion of catecholamines (CA) and serotonin (5-HT) into fluorescent compounds that could be visualized at the cellular level [18]. This new histochemical approach allowed for an accurate mapping of monoamine neurons in the brain [19,20,21], which was further expanded by the subsequent widespread introduction of immunohistochemical techniques leading to detailed maps of the distribution of various types of neurons on the basis of their transmitter substance [22,23]. On this basis, sets of tools designed to provide an exhaustive map in animals of the neurotransmitters and the neurons/circuits that express them (i.e., the so-called “chemoconnectome”) have been proposed [24]. In this context, fluorescent sensors to monitor neurotransmitters (such as acetylcholine) in vitro and in vivo, as well as suitable for epifluorescence, confocal, and/or two-photon microscopy, are under development [25]. In humans, imaging techniques, such as positron emission tomography [26] and mass spectrometry imaging [27], presently provide powerful tools for the comprehensive mapping of neurotransmitter networks in specific brain regions. 

**Figure 1:**
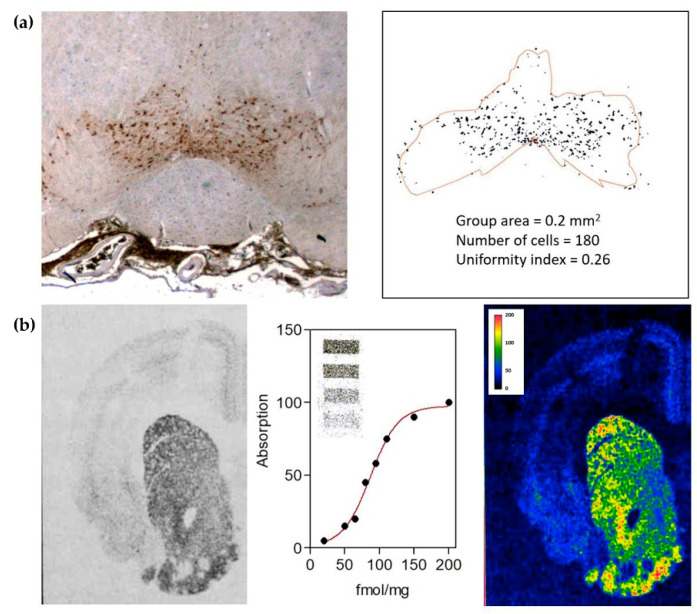
(**a**) Computer-assisted image analysis of a transmitter-identified nerve cell group. Dopaminergic cells in the rat ventral tegmental area are shown in the left panel. Automatic image analysis methods (see [29]) can be used to discriminate cell profiles and to estimate the tissue area populated by the cell group (contour line) in order to evaluate parameters characterizing the size of the cell group and the distribution of the cells in that area. (**b**) Computer-assisted image analysis of receptors. Dopamine receptor distribution in rat striatum (left panel) as visualized by in situ 3H-spiperone binding and autoradiography (see [35,36]). Image intensities can be calibrated in terms of the amount of binding by using standards of known concentrations of the ligand (middle panel). Thus, both the overall distribution and the number of receptors in the different regions of the tissue can be evaluated (right panel).

The impact of these observations significantly increased when applications of computer-assisted image analysis methods became available [6,28]. In fact, they allowed for the estimation not only of morphometric parameters suitable to quantitatively characterize populations of transmitter-identified neuronal profiles (cell bodies, dendrites, or nerve terminals) in morphological terms (Figure 1a), but also microdensitometric data providing an evaluation of the neurotransmitter content based on the staining intensity observed on histochemical or immunohistochemical preparations [29] processed according to well-standardized protocols [30,31]. Thus, by these methods, a quantitative description of changes transmitter-identified neuronal populations may undergo became possible, providing a tool to explore the possible roles of different neurotransmitters in the etiology of disease states and the efficacy at cellular level of therapeutic interventions. 

From the pharmacological research standpoint, the study of receptors is certainly a key component. The interplay between chemical neuroanatomy techniques and methods for the quantitative analysis of images (Figure 1b) significantly contributed to this investigation also, allowing the direct observation in CNS tissue of receptor distribution and expression levels [32], as well as the possibility to estimate in situ binding parameters (e.g., receptor affinities) through saturation binding assays [33] combined with microdensitometric evaluations [34]. In this respect, traditionally, these studies have used specific ligands labelled with radioisotopes [35,36]. However, due to safety concerns associated with exposure to radioactivity and the costs associated with licensing and disposal, a viable labelling substitute has been sought. Thus, more recently, a variety of fluorescence- and bioluminescence-based approaches have been developed to map receptor distribution and to measure ligand binding in situ [37,38,39].

A further form of WT is represented by gap junctions (GJ). GJ can be observed in electrical synapses (representing a minority of interneural connections in mammals) and seem to play a particular role in astrocytes, where they allow the coupling of astrocytes to each other to form networks [40,41] in which cells can exchange signals mediated by calcium waves [42]. In the last decades, this process was widely visualized and monitored in vitro by using fluorescent calcium indicators and quantitative microscopy techniques [43,44,45]. Tools for studying Ca^2+^ dynamics in vivo have also been developed [46], allowing for simultaneous image astrocyte calcium activity alongside astrocyte-specific perturbations (see [47]). Since the GJ channel can also be regulated by extra- and intracellular signals [48], it has been suggested that astrocyte networks could implement computational processes [42,49] and participate in higher brain functions [50,51]. 

Increasing evidence also indicates a direct involvement of astrocytes in the regulation of neuronal excitability and action potential propagation, as demonstrated by studies on excitatory synapses leading to the proposal of the concept of “tripartite synapse” [52]. According to this view, the communication between astrocytes and neurons is a bidirectional one, with neural activity influencing astrocytic activation, which in turn modulates the activity of neurons [53]. To monitor the extracellular environment, indeed, astrocytes express specific receptors and channels (see [54]). Notably, astrocytes can express many neurotransmitter receptors also expressed by neurons, allowing them to respond to a variety of neuronal signals [55]. Conversely, the induced calcium dynamics, mediated by gap junctions, can trigger the release of gliotransmitters (D-serine, ATP, glutamate) from astrocytes, leading to a direct regulation of ongoing neural activity [56,57]. As indicated by a number of experimental studies (reviewed in [53]), this intercellular crosstalk significantly influences both short- and long-term synaptic plasticity and, as a consequence, higher CNS functions such as learning and memory [53].

### 2.2. Volume Transmission

The VT proposal originated from morphological observations on transmitter–receptor mismatches mainly based on double immunolabelling experiments. As a matter of fact, the first paper on VT was based on the lack of correlation between the regional distribution of central enkephalin and beta-endorphin immunoreactive terminals and of opiate receptors [58]. This morphological arrangement was later observed in a number of other systems [59,60]. However, already in the first papers on VT, it was stressed that the mismatch phenomenon is a necessary but not sufficient condition to assess VT [61]. Thus, crucial aspects such as the existence of sources of VT signals, the existence of energy gradients allowing the VT signal migration, and of preferential pathways in the CNS have been investigated. 

In this respect, VT mainly employs the same set of signals as WT, namely, transmitters, peptides, ions, and gases (such as nitric oxide [62]). An important finding was that non-synaptic receptors providing the decoding mechanism for these signals are generally characterized by a higher affinity [63]. Other types of signals, however, were suggested to be exchanged by VT [64,65]. They include physical signals, such as pressure waves [66,67], field potentials modulating the electrical and chemical properties of the plasma membranes of the neighboring cells [68], and thermal waves, especially affecting neurons endowed with high Q10 values (i.e., sensitivity to temperature elevation), such as those present in particular hypothalamic regions [69]. Thus, decoding systems based on processes of membrane polarization and on chemical reactions [70] are also exploited by VT. In neurons, the release of VT signals may occur by spillover from the synaptic cleft or through extrasynaptic mechanisms from the soma, axons, and dendrites in the absence of postsynaptic counterparts (see [71] for a review). VT signals, however, are also released by glial cells [72] to reach a variety of targets within entire CNS regions and represent a key modulatory factor of synaptic activity [73].

VT is primarily mediated by simple diffusion but also by pressure waves due to the arterial pulses in the cerebral arteries and by thermal and electrical gradients [67]. Thus, VT often uses energy gradients that are also used for other goals (such as the renewal of the ECF). On this basis, it has been pointed out that generation of physical VT signals (such as pressure waves in cerebral arteries and temperature waves) and chemical VT signal migration are in many instances by-products of phenomena going on in the brain to fulfil other tasks. In other words, this mode of intercellular communication provides an example of a “tinkering” process [74].

With regard to the communication pathways, VT mainly exploits the several often spatially divergent tortuous channels [75,76] made by the clefts (about 20 nm in diameter) between cells and filled with extracellular fluid and extracellular matrix. Hyaluronan, tenascin, and lecticans (different types of chondroitin sulfate containing proteoglycans) are the major building blocks of the extracellular matrix [77] that via protein–protein and protein–carbohydrate interactions form a three-dimensional structure modulating the diffusion and flow pathways of VT. The size of the meshes and the degree of hydration will control the diffusion pathways in the ECS and thus VT [78]. ECS, therefore, fulfills active tasks in the transmission process since it may direct the diffusion of electrochemical messages in an anisotropic fashion, favoring or preventing the communication between two CNS areas. At least in part, this may be also due to the fact that the extracellular matrix is not an amorphous filler and can also differ among the various tissue regions, depending on the cell types populating them [79]. 

Nicholson’s work (see [80]) provided a deep characterization of the biophysical features of VT. Diffusion in the interstitial space was accurately modeled and the structure of the ECS was characterized by estimating specific morphometric parameters (e.g., volume fraction and tortuosity). To estimate these features, the development of specific quantitative techniques on CNS tissues played a key role. They involve indirect approaches, such as, for instance, the real-time iontophoresis with tetramethylammonium [81], as well as more direct methods based on fluorescent dyes [82].

## 3. Mechanisms Modulating Intercellular Communication in the CNS

The view on intercellular communication in the CNS was significantly expanded when it clearly appeared that both signaling, and decoding processes can be finely modulated by a variety of mechanisms of potential great interest also from a pharmacological standpoint. They will be the focus of the sections that follows. 

### 3.1. Coexistence of Messengers

With regard to synaptic transmission, an historical principle in neuroscience is the so-called Dale’s principle, stating that a given neuron contains and releases only one neurotransmitter and exerts the same functional effects at all of its termination sites [83,84]. An important progress was the demonstration that the original view of the synapse, as proposed according to the Dale’s principle, was not the whole truth [85]. Several authors (see [86,87]), indeed, gave clear demonstrations that one and the same neuron can synthesize more than one neurotransmitter. 

Dual labeling, immunohistochemical techniques represent the key tool to directly demonstrate the co-localization of substances in the same cell, leading to a plethora of studies showing multiple transmitters in single synaptic terminals [88,89]. As illustrated in Table 1, neuropeptides represent the largest class of neurotransmitters commonly present as cotransmitters [90].

**Figure 2:**
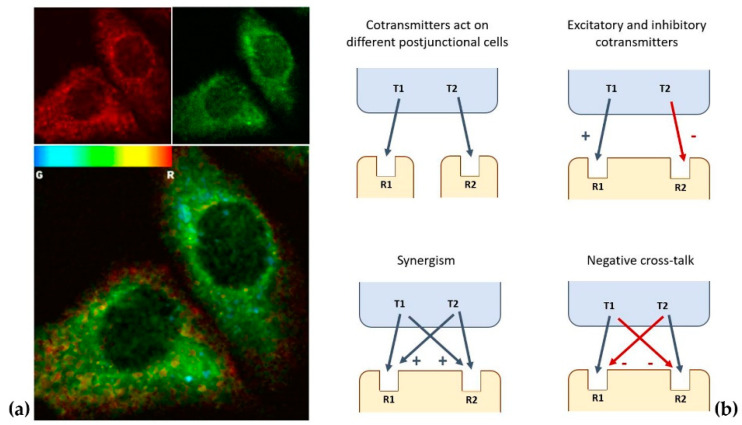
(**a**) Immunofluorescence images of cholecystokinin (red fluorescence) and dopamine (green fluorescence) in cultured nerve cells. In the lower panel, the corresponding map of green/red ratios is shown, from which the degree of co-localization can be evaluated by computer-assisted image analysis protocols (see [105] for details). (**b**) Examples of signaling variations offered by co-transmission (see [110] for details).

Specific computer-assisted image analysis approaches, designed to detect and characterize staining co-localization (see Figure 2a) make a quantitative description of this microanatomical feature also possible [105,106,107].

Cotransmitters have been defined in different ways, for example, slow/fast, ionotropic/metabotropic, or conventional/modulatory (see [108] for a terminological discussion). However, there are exceptions to the various classification schemes. We know, indeed, that the single substances can serve different roles depending on where and when they are released and the receptors to which they bind. For instance, even if the neuronal cell synthetizes more than one neurotransmitter, processes have been described in which different neurotransmitters are segregated to different presynaptic terminals facing distinct excitatory or inhibitory postsynaptic sites [109]. 

In this respect, some general aspects of cotransmission can be outlined (see [110] for a detailed recent review). Firstly, co-localization and release of multiple transmitters provides functional flexibility to neuronal circuits that are anatomically hard-wired [111], increasing neuronal signaling repertoire (see Figure 2b for some examples). Furthermore, data exist suggesting that released cotransmitters could be involved in longer-distance VT as a mechanism working in parallel with conventional WT [65]. Thus, a single transmitter can diverge to affect multiple receptors on multiple targets, while multiple transmitters can converge onto single effectors [112,113], and the effects can change depending on the functional state of the targets [110].

With the continual development of stimulation and imaging techniques of ever-increasing resolution, the impact of cotransmission on CNS activity has blossomed [114,115], and mechanisms characterizing cotransmission (such as convergent and divergent cotransmission, different temporal dynamics of ionotropic and metabotropic cotransmission, and focal regulation of cotransmitter release) have been characterized in vivo, together with diverse new ways in which activity is modified by cotransmission [110].

### 3.2. Receptor–Receptor Interactions

Mechanisms modulating the decoding apparatus are present at the post-synaptic level. It is well known, for instance, that a single postsynaptic side may have more than one kind of receptor for a given transmitter, with each receptor type controlling a different chemical decoding mechanism or ionic conductance channel [116]. Thus, the synapse can be endowed with multiple signal transduction lines, each represented by a neurotransmitter and a corresponding set of different receptors. Furthermore, receptors can functionally interact by sharing signaling pathways or by mechanisms of transactivation [117,118].

In the 1980s, however, by means of in vitro and in vivo experiments, Agnati, Fuxe, and collaborators provided indirect biochemical and functional evidence that G-protein-coupled receptors (GPCRs) could also form macromolecular complexes at the membrane level [119,120] as a consequence of direct allosteric receptor–receptor interactions (RRI). The term RRI refers to an interaction requiring a direct physical contact between the involved receptor proteins, leading to the formation of receptor complexes (dimers or high-order oligomers) at the cell membrane (see [121] for the definition, as assessed by a specific international consensus workshop). The concept of GPCR oligomerization was later confirmed by studies reporting that two nonfunctional class C GPCR monomers, GABA-B1 and GABA-B2, assembled in a signaling heterodimer [122], and in the years that followed, several groups provided direct evidence for the existence of receptor complexes formed by GPCRs [123,124,125,126,127,128,129,130,131,132,133]. 

**Figure 3:**
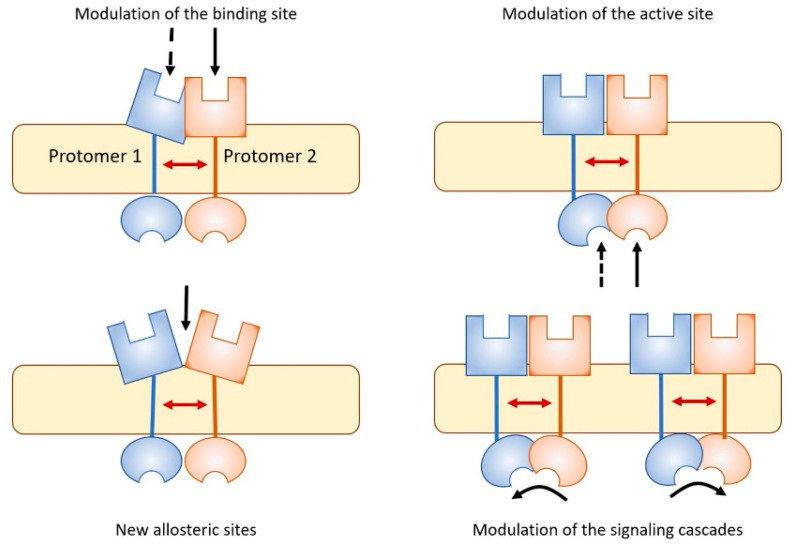
As a result of allosteric RRI, receptor complexes appear to be endowed with pharmacological features that cannot be fully derived from the characteristics of the single participating protomers (see [140] for a review). Some examples are illustrated. Complex formation can lead to modulation of the binding sites, modulation of intracellular active sites, generation on the complex of novel sites suitable for the binding of allosteric modulators, and modulation of the signaling cascade, as for instance the switch from G protein to β-arrestin signaling in GPCRs.

The basic biochemical mechanism leading to the formation of these receptor assemblies are allosteric interactions, and, as outlined by Changeux and Christopoulos [134], the cooperativity that emerges in the actions of orthosteric and allosteric ligands of the GPCRs forming the assembly provides the cell decoding apparatus with sophisticated dynamics (see [135,136,137] for reviews) in terms of modulation of recognition, G-protein signaling and selectivity, receptor desensitization [138], and switching to β-arrestin signaling [139]. Figure 3 schematically illustrates examples of these processes.

**Figure 4:**
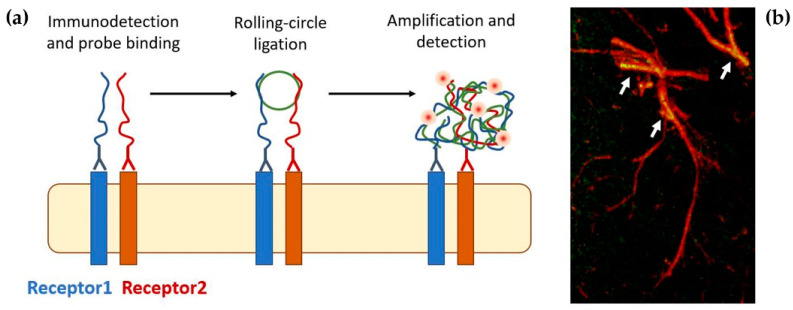
(**a**) Schematic of the PLA reaction (see [143,144]). In a first step, the proteins of interest are recognized by specific antibodies coupled to oligonucleotides (PLA probes). Next, hybridizing connector oligos join the PLA probes only if they are in close proximity to each other (<10 nm), and ligase forms a closed-circle DNA template that is required for rolling-circle amplification in which the resulting closed, circular DNA template becomes amplified by DNA polymerase. Lastly, complementary detection oligos coupled to fluorochromes hybridize to repeating sequences in the resulting oligonucleotide complex, allowing the visualization of the interacting proteins. (**b**) PLA signals are detected by fluorescence microscopy as discrete spots and provide the cellular localization of the interacting receptors. The image shows the detection of adenosine A_2A_-dopamine D_2_ heteroreceptor complexes in astrocyte processes [147].

Diverse methods are presently available for identifying direct interactions between GPCRs. Of particular interest for the present discussion are biophysical proximity assays, being based on the combination of histochemical techniques and quantitative microscopy. They involve resonance energy transfer methods [141], line-scan fluorescence cross-correlation spectroscopy [142], and proximity ligation assay (see [143,144] for details). Proximity ligation assay (PLA, illustrated in Figure 4) is a fluorescence-based approach of particular interest since it makes possible the study of the localization and modulation of heteroreceptor complexes as fixed tissue is used. A novel sensitive approach, based on the use of the AlphaScreen technology [145] to detect GPCR heteromerization even under nonoptimal conditions, has been very recently proposed by Fernández-Dueñas and collaborators [146]. It allowed the authors to reveal heteromers in human post-mortem brains from healthy and Parkinson’s disease subjects.

Receptor complexes have been observed in both neurons and glial cells (see [148] for a recent review), and oligomeric organization represents a quite common feature in all the receptor families, with the ion channel receptors (where multimerization is needed) lying at one end of the spectrum and GPCRs at the other [140]. Thus, oligomerization emerges as an efficient mechanism for tuning the functionality of receptor proteins, including those able to signal as monomers, such as GPCRs [134]. In this respect, recently reported evidence for receptor complexes involving protomers from different families [140,149] is also of significant interest.

More generally, a pattern of interactions between molecules embedded and/or associated with the cell membrane has been observed, leading to the formation of the so-called horizontal molecular networks (HMN) that can operate as complex modules carrying out specialized tasks [150]. 

In this respect, a first aspect deserving consideration is the lipid environment. It was shown to influence receptor function and several health disorders during aging were assigned to changes in the membrane composition that altered receptor signaling [151]. Furthermore, several membrane proteins specifically interacting with receptors have been identified. For instance, already 50 or more GPCR-interacting proteins (GIP) have been demonstrated [152], mostly serving as scaffolding or adapter proteins [153,154]. Thus, receptors and receptor complexes are in the center of multiple receptor–protein and protein–protein interactions that can influence their function and assemblage (see [155]).

### 3.3. Intercellular Exchange of Components of the Signalling Apparatus

A possible peculiar mode of VT was called the Roamer-Type of VT (RT-VT) [7] and involves the exchange of a set of chemical messages via extracellular vesicles acting as protective containers [156]. Different types of micro-vesicles (MV) have been described (see [7]), but two types are of major importance for our discussion. Exosomes are vesicles (40–100 nm in diameter) contained in the so-called early, late, or recycling endosomes, types of multivesicular bodies [157]. Extracellular vesicles can also be formed from lipid raft domains of the plasma membrane and are then called shedding vesicles [158]. Thus, shedding vesicles show surface markers that are dependent on the composition of the membrane of origin and constitute a larger and more heterogeneous population of extracellular vesicles, ranging from 100 to 1000 nm in diameter.

The possible relevance of this type of VT for the intercellular communication can be appreciated in the light of several experimental findings. A number of receptors, indeed, were found to be transferred from one to another cell type via the exosome pathway [159]. Regarding GPCR involved in the recognition/decoding of signals between CNS cells, recent data were obtained, demonstrating that they can be transported between neurons by MV and in particular by exosomes [160,161]. Thus, the available experimental evidence suggests that this mode of intercellular communication can lead to the transient acquisition by the target cell of a new phenotype, enabling it to recognize/decode transmitters and/or modulators for which the cell does not express the pertinent cognate receptors. 

This type of VT was found to be exploited by astrocytes as well [162], and it is noteworthy that the exosomes released from the astrocyte processes proved the ability to selectively target neurons. 

MV release in the roamer type of VT can therefore represent a novel mechanism for the modulation of neuron–neuron and glia–neuron intercellular communication.

## 4. Present Views on Intercellular Communication in the CNS may Impact on Neuropharmacology

The views on CNS structure and function emerging from the intermingling of chemical anatomy and quantitative microscopy may significantly contribute to the development of new pharmacological approaches with a major impact on molecular medicine.

Neurotransmission, indeed, is central to neuropharmacology. Many drugs are presently available to modulate disease states of the CNS by acting on neurotransmission processes (see [163]). Some drugs (e.g., prescription opioids) mimic neurotransmitters to engage and stimulate their specialized receptors. Others alter neurotransmission by interfering with processes of synthesis, incorporation into vesicles or metabolism of some neurotransmitter. Finally, most drugs are basically agonists or antagonists of receptors. In this context, quantitative chemical neuroanatomy techniques contributed to monitoring the efficacy of the applied protocols and their side effects as well. An example is provided by early studies based on receptor binding autoradiography allowing for the characterization of dopamine receptor supersensitivity in striatum (a condition inducing diskinesia) following treatment with the neuroleptic drug haloperidol [164].

However, current pharmacological approaches for neurological disorders often have limited efficacy at best. There could be many reasons for this limited efficacy, but the most obvious is that most pharmacological approaches do not mimic the normal endogenous release of cotransmitters or neurotransmitter interactions, and thus the normal chemical environment of the relevant circuits [110]. In this respect, the new views on the intercellular communication in the CNS emerged in the last decades from the studies based on quantitative chemical neuroanatomy approaches, potentially allowing for the proposal of new perspectives to target the problem. In particular, mechanisms modulating the neurotransmitter signaling (see Section 2) may open new possibilities to pharmacology.

### 4.1. Targeting Cotransmitters

Although our understanding of the functional effects of co-localized neurotransmitters in higher functions is still in its infancy [110], research efforts aimed at exploiting this feature from a pharmacological standpoint are underway. An example is provided by studies on L-dopa-induced dyskinesia (LID), a debilitating side effect of dopamine replacement therapy for Parkinson’s disease (PD). Studies in animals with complete lesions of the nigrostriatal dopamine pathway showed that LID is critically dependent on the integrity and function of the serotoninergic system [165] since dampening of serotonin neuron activity by drugs resulted in a near-complete block of LID. Serotonin neurons, indeed, are able to convert exogenous L-dopa into DA and release it as a “false cotransmitter” in a non-physiological “dysregulated” manner. More recently, a role for a true cotransmitter located in mesencephalic dopaminergic neurons has been described [166]. It indicated that diminished sonic hedgehog (Shh) signaling in the basal ganglia, caused by the degeneration of midbrain dopamine neurons, facilitates the formation and expression of LID and that pharmacologically augmenting Shh signaling in the L-Dopa-treated brain may be a promising therapeutic approach for mitigating the dyskinetic side effects of long-term treatment with L-dopa. A mechanism by which antipsychotic drugs may alter the dynamics between dopamine and co-localized peptides has also been described and may be relevant to the pharmacotherapy of schizophrenia [167]. It is based on the intrinsic ability of these agents to stimulate dopamine neuronal activity while blocking dopamine receptors. This feature modulates the ratio of catecholaminergic to peptidergic transmission within the mesotelencephalic system, leading to imbalances of peptide and dopamine cotransmission.

### 4.2. Targeting Volume Transmission

VT processes also deserve consideration in neuropharmacology. From one side, it is relevant for the pharmacokinetics and actions of neuropsychoactive drugs, since it has been observed [168] that they can be regarded as exogenous VT signals in that they diffuse in the cerebral extracellular space and are constrained there by the same factors that influence migration of endogenous VT signals. On the other side, increasing evidence indicates the involvement of endogenous VT signals in CNS imbalance and disease. Nitric oxide [169], oxytocin [170], melanin-concentrating hormone [171], and CNTF [172] provide some examples. It has also been suggested [173] that in neuroinflammation, glial microvesicles may be released, containing chemokine and cytokine receptors. The extracellular vesicles reaching by roamer-type of VT surrounding neurons can be internalized via cell adhesion receptors, contributing to positive, negative, and/or cognitive symptoms of schizophrenia in line with the mild encephalitis hypothesis of this pathology [174]. 

In the dorsal striatum, dopamine diffusion can modulate the different striatal nerve cell types by acting on extrasynaptic dopamine receptor subtypes and receptor complexes in order to provide its unique and crucial fine-tuning of movements, which is lost in PD [175]. Thus, targeting non-synaptic receptors and receptor complexes can represent a useful pharmacological approach to balance wiring and volume transmission in selected circuits [176].

### 4.3. Targeting Receptor Complexes

Receptor complexes emerging from allosteric RRI likely represent the most explored field to design new pharmacological strategies for the treatment of CNS pathologies (see [135]). In the last decade, such a research effort allowed for the characterization of a panel of receptor complexes representing possible targets for the treatment of pathologic conditions, such as Parkinson’s disease [177], schizophrenia and depression [173,178], neuropathic pain [179], addiction [180], and food intake disorders [181]. On this basis, novel strategies for drug treatment have also been proposed. The most followed approach to target receptor complexes was the use of agonists/antagonists of a given protomer, which is based on the fact that the pharmacology of some agonists/antagonists of a given protomer in terms of affinity and efficacy may show substantial differences among various types of receptor complexes.

Interestingly, the tested pharmacological protocols when compared to the traditional ones often appeared able to reduce collateral effects [182]. An example of this type of approach is provided by studies (reviewed in [183]) on the heteromer formed by adenosine A_2A_ (A2A) and dopamine D_2_ (D2) receptors, highly concentrated in the striatum, mainly in the nerve terminals of GABAergic enkephalinergic neurons [184]. A2A–D2 heteroreceptor complexes were first demonstrated in cellular models by BRET/FRET techniques [185], then confirmed in membrane preparations by atomic force microscopy [186] and in tissues by PLA [143]. Antagonistic A2A–D2 interactions occur in the heterodimer, as demonstrated by receptor autoradiography studies showing a reduction of the affinity of the high-affinity D2-agonist-binding site after A2A receptor agonist treatment in the nucleus accumbens core and shell of rats and humans [187]. Thus, the A2A receptor in the heteroreceptor complexes localized in the dorsal striatopallidal GABA neurons became a major target for a strategy based on the use of A2A receptor antagonists in treatment of PD. Animal models of PD in nonhuman primates and rodents [188] gave support to this hypothesis; clinical data on this therapeutic strategy (see [189] for a review) were also obtained; and, in this respect, it is of interest to mention the recent approval in the United States of an A2A antagonist (istradefylline) as an adjunctive treatment to L-DOPA in patients with PD experiencing “off” episodes [190]. Alternative approaches to design receptor-complex-selective compounds are also under study. They involve the identification on the resulting quaternary structure of allosteric sites suitable for the binding of some modulator [191], or the development of receptor-complex-specific bivalent ligands [192].

### 4.4. Targeting Glial Cells

As discussed in previous sections, recent evidence pointed to the significant involvement of glia–glia and glia–neuron communication in modulating and shaping synaptic activity and plasticity [47,53], leading to a deeper understanding of how glial cells contribute to information processing within the neural circuitry [54].

Astrocytes, as abovementioned, are capable of release of gliotransmitters [193] such as ATP [194] and D-serine [195]. They are characterized by a polarized orientation of their processes [196]. While one or only few processes have contacts with CNS boundaries such as capillaries and pia, an overwhelming number of thin filopodia- and lamellipodia-like process terminals contact and enwrap synapses. Thus, the function and efficacy of synaptic transmission are determined not only by the composition and activity of pre- and postsynaptic components but also by the features of the astrocytic processes that enwrap the synapse. 

Recently, it has been shown that microglia cells are also active players in regulating synaptic development and plasticity in the brain [197]. However, the way in which they influence the normal functioning of synapses is still largely unknown. 

As mentioned before, any type of glial cell can release VT signals, including extracellular vesicles of different kinds, both in physiologic and pathological conditions [198]. Furthermore, they express a large panel of receptors to monitor the extracellular environment and receptor complexes have been identified on glial cells (see [148]). 

Pharmacological research specifically applied to glial receptor complexes is still beginning. However, it may represent a topic of particular interest from a therapeutic standpoint. Indeed, as suggested by some of the studies here mentioned, it opens the possibility to explore novel, glia-mediated strategies to address neurodegenerative [199] and functional [200] CNS disorders.

## 5. Concluding Remarks

Most of the new evidence that has been acquired in the last decades on intercellular communication in the CNS is a paradigmatic case of the scientific jumps that are made possible by the synergic interactions of different disciplines. In particular, the synergic interactions between chemical neuroanatomy approaches and quantitative analysis of the obtained images have produced a deeply different view on brain structure and functions. This research effort, indeed, showed that the anatomical mapping of the relationship among CNS components significantly contributes to a deeper level of understanding of CNS functions, indicating that CNS accomplishes its activity mainly through the integrative actions of networks in which functions emerge from collections of elementary units (nodes) that interact dynamically [201]. This was a turning point that allowed for extraordinary investigations on brain circuits, leading to the development of a new discipline, namely, “brain connectomics” [8,9]. 

Quantitative biochemical anatomy approaches, however, also suggested that CNS functions may result from the integration of the information handling at different miniaturization levels. As a matter of fact, CNS structure appears organized according to a hierarchical, nested, architecture arranged like a “Russian doll” [9,202], where macroscale (i.e., brain areas), mesoscale (i.e., sub-circuits involving neurons and glial cells), microscale (single cells and intercellular connections, such as synapses), and nanoscale (molecular networks and receptor complexes) can be defined as crucial levels of organization. As discussed in the previous sections, this new view allowed for a deeper characterization of CNS structure and function in both physiological and pathological conditions, opening new possibilities for the development of novel pharmacological, surgical, and physical (e.g., in situ electro-chemical stimulations) approaches finalized at overcoming crucial deficits in the CNS circuits [203,204]. 

Several aspects, however, remain to be addressed. A first point concerns cotransmission (see [110]). To better understand the functional effects of cotransmission, we would need to examine and mimic in vivo release and we also need to better understand the signals carried by the interaction of various cotransmitters. In this respect, optogenetic techniques [205,206] combined with exogenous application of substances could provide tools facilitating the analysis of these aspects. A second significant line of research concerns receptor complexes resulting from RRI at the cell membrane. During the last 20 years, a large number of GPCRs have been reported to form homodimers, heterodimers, and higher order oligomers (see [136,137,207]), while data on mapping and characterization of receptor complexes involving receptors from different families are more limited [149,208]. The dynamics of formation and dissociation of receptor complexes is also a topic of significant interest to have a deeper understanding of their role. Class A GPCRs, for instance, are often transient structures, characterized by a monomer–dimer equilibrium in the cell membrane [209]. Microanatomical techniques such as time-resolved fluorescence may be of help to unravel this feature [210]. As discussed previously, the search for heteromer-selective drugs is a field of research gaining great attention. In this context, a topic of possible development would also be the identification of pharmacological tools separately targeting synaptic and extrasynaptic receptors [211,212] in order to design strategies to rebalance WT and VT. Finally, a direction of future research is certainly targeting glial cells as a strategy to treat neurological disorders. The abovementioned intimate association of glia with neurons, indeed, is at the basis of increasing evidence that metabolic perturbations of glial cells may alter neuron–glial interactions, potentiating the underlying pathology of many neurological diseases [213]. However, the mechanism driving the circumstantial activation of glial phenotypes is just starting to unravel, and future studies should open new perspectives.

The future, therefore, may provide new insights in the intercellular communication in the CNS, opening new frontiers for the development of novel therapeutic approaches.

## Figures and Tables

**Table 1:** Co-localized transmitters in CNS regions.

Region	Species	Co-Localized Transmitters	Reference
Medulla oblongata	human, rat	Noradrenaline, neuropeptide Y	[91]
	rat	Adrenaline, neuropeptide Y	[91]
	rat	Adrenaline, neurotensin	[92]
	rat, cat	Serotonin, substance P	[93]
	rat	Serotonin, thyrotropin releasing hormone	[93]
	cat	Serotonin, enkephalin	[94]
Pons	cat	Serotonin, enkephalin	[94]
	rat	Acetylcholine, substance P	[95]
Locus coeruleus	cat	Noradrenaline, enkephalin	[96]
	rat	Noradrenaline, ATP	[97]
Ventral tegmental area	rat	Dopamine, neurotensin	[92]
	human, rat	Dopamine, cholecystokinin	[98]
Hypothalamus	rat	Noradrenaline, ATP	[97]
	mouse	GABA, ATP	[99]
Thalamus	cat	GABA, somatostatin	[100]
Hippocampus	rat	Glutamate, ATP	[101]
Caudate nucleus	rat	Acetylcholine, ATP	[102]
Mesolimbic system	rat	Dopamine, ATP	[103]
Cortex	rat	Acetylcholine, vasoactive intestinal peptide	[104]
	rat	Acetylcholine, ATP	[102]
Retina	mouse	GABA, ATP	[99]

## Data Availability

Data sharing is not applicable since no new data were recorded or analyzed in this study.

## References

[1] Mazzarello P. (2010). Golgi: A Biography of the Founder of Modern Neuroscience.

[2] Ramón y Cajal S. Nobel Lecture. www.nobelprize.org/prizes/medicine/1906/cajal/lecture/.

[3] Golgi C. Nobel Lecture. www.nobelprize.org/prizes/medicine/1906/golgi/lecture/.

[4] Sherrington C.S. (1906). The Integrative Action of the Nervous System.

[5] Series: Handbook of Chemical Neuroanatomy.

[6] Agnati L.F., Fuxe K. (1985). Quantitative Neuroanatomy in Transmitter Research.

[7] Agnati L.F., Guidolin D., Maura G., Marcoli M., Leo G., Carone C., De Caro R., Genedani S., Borroto-Escuela D.O., Fuxe K. (2014). Information handling by the brain: Proposal of a new “paradigm” involving the roamer type of volume transmission and the tunneling nanotube type of wiring transmission. J. Neural Transm..

[8] Sporns O. (2012). Discovering the Human Connectome.

[9] Guidolin D., Marcoli M., Maura G., Agnati L.F. (2017). New dimensions of connectomics and network plasticity in the central nervous system. Rev. Neurosci..

[10] Kuhn T. (2012). The Structure of Scientific Revolutions: 50th Anniversary Edition.

[11] Agnati L.F., Guidolin D., Guescini M., Genedani S., Fuxe K. (2010). Understanding wiring and volume transmission. Brain Res. Rev..

[12] Guillemin R. (1978). Peptides in the brain: The new endocrinology of the neuron. Science.

[13] Nicholson C., Schmitt F.O., Worden F.G. (1979). Brain cell microenvironment as a communication channel. The Neurosciences: Fourth Study Program.

[14] Schmitt F.O. (1984). Molecular regulators of brain function. A new view. Neuroscience.

[15] Vizi E.S. (1980). Non-synaptic modulation of transmitter release: Pharmacological implication. Trends Pharmacol. Sci..

[16] Kavalali E.T. (2015). The mechanisms and functions of spontaneous neurotransmitter release. Nat. Rev. Neurosci..

[17] Rangel-Gomez M., Meeter M. (2016). Neurotransmitters and novelty: A systematic review. J. Psychopharmacol..

[18] Falck B., Hillarp N.A., Thieme G., Torp A. (1962). Fluorescence of catecholamines and related compounds condensed with formaldehyde. J. Histochem. Cytochem..

[19] Fuxe K. (1963). Cellular localization of monoamines in the median eminence and in the infundibular stem of some mammals. Acta Physiol. Scand..

[20] Dahlström A., Fuxe K. (1964). A method for the demonstration of monoamine-containing nerve fibres in the central nervous system. Acta Physiol. Scand..

[21] Andén N.E., Carlsson A., Dahlström A., Fuxe K., Hillarp N.A., Larsson K. (1964). Demonstration and mapping out of nigro-neostriatal dopamine neurons. Life Sci..

[22] Hökfelt T., Johansson O., Goldstein M. (1984). Chemical anatomy of the brain. Science.

[23] Yelnik J., Bardinet E., Dormont D., Malandain G., Ourselin S., Tandé D., Karachi C., Ayache N., Cornu P., Agid Y. (2007). A three-dimensional, histological and deformable atlas of human basal ganglia. I. Atlas construction based on immunohistochemical and MRI data. NeuroImage.

[24] Deng B., Li Q., Liu X., Cao Y., Li B., Qian Y., Xu R., Mao R., Zhou E., Zhang W. (2019). Chemoconnectomics: Mapping Chemical Transmission in Drosophila. Neuron.

[25] Jing M., Zhang P., Wang G., Feng J., Mesik L., Zeng J., Jiang H., Wang S., Looby J.C., Guagliardo N.A. (2018). A genetically encoded fluorescent acetylcholine indicator for in vitro and in vivo studies. Nat. Biotechnol..

[26] Ceccarini J., Liu H., Van Laere K., Morris E.D., Sander C.Y. (2019). Methods for quantifying neurotransmitter dynamics in the living brain with PET imaging. Front. Physiol..

[27] Shariatgorji M., Nilsson A., Fridjonsdottir E., Vallianatou T., Källback P., Katan L., Sävmarker J., Mantas I., Zhang X., Bezard E. (2019). Comprehensive mapping of neurotransmitter networks by MALDI-MS imaging. Nat. Meth..

[28] Zoli M., Guidolin D., Agnati L.F. (1992). Morphometric evaluation of populations of neuronal profiles (cell bodies, dendrites, and nerve terminals) in the central nervous system. Microsc. Res. Tech..

[29] Zoli M., Zini I., Agnati L.F., Guidolin D., Ferraguti F., Fuxe K. (1990). Aspects of neural plasticity in the central nervous system—I. Computer-assisted image analysis methods. Neurochem. Int..

[30] Grube D. (2004). Constants and variables in immunohistochemistry. Arch. Histol. Cytol..

[31] Bernardo V., Lourenço S.Q.C., Cruz R., Monteiro-Leal L.H., Silva L.E., Camisasca D.R., Farina M., Lins U. (2009). Reproducibility of immunostaining quantification and description of a new digital image processing procedure for quantitative evaluation of immunohistochemistry in pathology. Microsc. Microanal..

[32] Davenport A.P., Kuc R.E. (2005). Immunocytochemical Localization of Receptors Using Light and Confocal Microscopy with Application to the Phenotypic Characterization of Knock-Out Mice. Methods Mol. Biol..

[33] Pollard T.D. (2010). A guide to simple and informative binding assays. Mol. Biol. Cell.

[34] Benfenati F., Cimino M., Agnati L.F., Fuxe K. (1986). Quantitative autoradiography of central neurotransmitter receptors: Methodological and statistics aspects with special reference to computer-assisted image analysis. Acta Physiol. Scand..

[35] Agnati L.F., Fuxe K., Benfenati F., Zini I., Zoli M., Fabbri L., Härfstrand A. (1984). Computer assisted morphometry and microdensitometry of transmitter identified neurons with special reference to the mesostriatal dopamine pathway—I. Methodological aspects. Acta Physiol. Scand. Suppl..

[36] Moyse E., Krantic S.M. (2000). Visualization of Receptors In Situ. Applications of Radioligand Binding.

[37] Sridharan R., Zuber J., Connelly S.M., Mathew E., Dumont M.E. (2014). Fluorescent approaches for understanding interactions of ligands with G protein coupled receptors. Biochim. Biophys. Acta.

[38] Stoddart L.A., White C.W., Nguyen K., Hill S.J., Pfleger K.D.G. (2016). Fluorescence- and bioluminescence-based approaches to study GPCR ligand binding. Br. J. Pharmacol..

[39] Allikalt A., Laasfeld T., Ilison M., Kopanchuk S., Rinken A. (2021). Quantitative analysis of fluorescent ligand binding to dopamine D_3_ receptors using live-cell microscopy. FEBS J..

[40] Carmignoto G. (2000). Reciprocal communication systems between astrocytes and neurones. Progr. Neurobiol..

[41] Lallouette J., De Pittà M., Ben-Jacob E., Berry H. (2014). The topology of astrocyte networks controls the propagation of intercellular calcium waves. BMC Neurosci..

[42] Pereira A., Furlan F.A. (2010). Astrocytes and human cognition: Modeling information integration and modulation of neuronal activity. Progr. Neurobiol..

[43] Milani D., Malgaroli A., Guidolin D., Fasolato C., Skaper S.D., Meldolesi J., Pozzan T. (1990). Ca^2+^ channels and intracellular Ca^2+^ stores in neuronal and neuroendocrine cells. Cell Calcium.

[44] Okubo Y., Iino M. (2020). Visualization of astrocytic intracellular Ca^2+^ mobilization. J. Physiol..

[45] Tang F., Wu C., Zhai Z., Wang K., Liu X., Xiao H., Zhuo S., Ping L., Tang B. (2022). Recent progress in small-molecule fluorescent probes for endoplasmic reticulum imaging in biological systems. Analyst.

[46] Tran C.H.T. (2022). Toolbox for studying neurovascular coupling in vivo, with a focus on vascular activity and calcium dynamics in astrocytes. Neurophotonics.

[47] Lyon K.A., Allen N.J. (2022). From synapses to circuits, astrocytes regulate behavior. Front. Neural Circ..

[48] Glaume C. (2010). Astroglial wiring is adding complexity to neuroglial networking. Front. Neuroenerg..

[49] Guidolin D., Albertin G., Guescini M., Fuxe K., Agnati L.F. (2011). Central nervous system and computation. Q. Rev. Biol..

[50] Robertson J.M. (2002). The astrocentric hypothesis: Proposed role of astrocytes in consciousness and memory formation. J. Physiol..

[51] Volterra A., Meldolesi J. (2005). Astrocytes, from brain glue to communication elements: The revolution continues. Nat. Rev. Neurosci..

[52] Araque A., Parpura V., Sanzgiri R.P., Haydon P.G. (1999). Tripartite synapses: Glia, the unacknowledged partner. Trends Neurosci..

[53] Sancho L., Contreras M., Allen N.J. (2021). Glia as sculptors of synaptic plasticity. Neurosci. Res..

[54] Nedergaard M., Verkhratsky A. (2012). Artifact versus reality-How astrocytes contribute to synaptic events. Glia.

[55] Allen N.J. (2014). Astrocyte Regulation of Synaptic Behavior. Annu. Rev. Cell Dev. Biol..

[56] Fellin T., Carmignoto G. (2004). Neurone-to-astrocyte signalling in the brain represents a distinct multifunctional unit. J. Physiol..

[57] Caudal L.C., Gobbo D., Scheller A., Kirchhoff F. (2020). The paradox of astroglial Ca^2+^ signals at the interface of excitation and inhibition. Front. Cell. Neurosci..

[58] Agnati L.F., Fuxe K., Zoli M., Ozini I., Toffano G., Ferraguti F. (1986). A correlation analysis of the regional distribution of central enkephalin and beta endorphin immunoreactive terminals and of opiate receptors in adult and old male rats. Evidence for the existence of two main types of communication in the central nervous system: The volume transmission and the wiring transmission. Acta Physiol. Scand..

[59] Fuxe K., Bunnemann B., Aronsson M., Tinner B., Cintra A., von Euler G., Agnati L.F., Nakanishi S., Ohkubo H., Ganten D. (1988). Pre- and postsynaptic features of the central angiotensin systems. Indications for a role of angiotensin peptides in volume transmission and for interactions with central monoamine neurons. Clin. Exp. Hypertens. A.

[60] Fuxe K., Rivera A., Jacobsen K.X., Höistad M., Leo G., Horvath T.L., Staines W., De la Calle A., Agnati L.F. (2005). Dynamics of volume transmission in the brain. Focus on catecholamines and opioid peptide communication and the role of uncoupling protein 2. J. Neural Transm..

[61] Fuxe K., Agnati L.F. (1991). Volume Transmission in the Brain, Novel Mechanisms for Neural Transmission.

[62] Steinert J.R., Chernova T., Forsythe I.D. (2010). Nitric oxide signaling in brain function, dysfunction, and dementia. Neuroscientist.

[63] Vizi E.S. (2000). Role of high-affinity receptors and membrane transporters in nonsynaptic communication and drug action in the CNS. Pharmacol. Rev..

[64] Agnati L.F., Cortelli P., Biagini G., Bjelke B., Fuxe K. (1994). Different classes of volume transmission signals exist in the central nervous system and are affected by metabolic signals, temperature gradients and pressure waves. Neuroreport.

[65] Fuxe K., Dahlström A., Jonsson G., Marcellino D., Guescini M., Dam M., Manger P., Agnati L.F. (2010). The discovery of central monoamine neurons gave volume transmission to the wired brain. Prog. Neurobiol..

[66] Krimer L.S., Muly E.C., Williams G.V., Goldman-Rakic P.S. (1998). Dopaminergic regulation of cerebral cortical microcirculation. Nat. Neurosci..

[67] Agnati L.F., Genedani S., Lenzi P.L., Leo G., Mora F., Ferré S., Fuxe K. (2005). Energy gradients for the homeostatic control of brain ECF composition and for VT signal migration: Introduction of the tide hypothesis. J. Neural Transm..

[68] Bullock T.H. (1997). Signals and signs in the nervous system: The dynamic anatomy of electrical activity is probably information-rich. Proc. Natl. Acad. Sci. USA.

[69] Rivera A., Agnati L.F., Horvath T.L., Valderrama J.J., de La Calle A., Fuxe K. (2006). Uncoupling protein 2/3 immunoreactivity and the ascending dopaminergic and noradrenergic neuronal systems. Relevance for volume transmission. Neuroscience.

[70] Yablonskiy D.A., Ackerman J.J., Raichle M.E. (2000). Coupling between changes in human brain temperature and oxidative metabolism during prolonged visual stimulation. Proc. Natl. Acad. Sci. USA.

[71] Del-Bel E., De- Miguel F. (2018). Extrasynaptic neurotransmission mediated by exocytosis and diffusive release of transmitter substances. Front. Syn. Neurosci..

[72] Parpura V., Zorec R. (2008). Exocytotic release from astrocytes. Brain Res. Rev..

[73] Gundersen V., Storm-Mathiesen J., Bergersen L.H. (1995). Neuroglial transmission. J. Physiol. Rev..

[74] Jacob F. (1977). Evolution and tinkering. Science.

[75] Jansson A., Maze L.T., Andbjer B., Rosen L., Guidolin D., Zoli M., Syková E., Agnati L.F., Fuxe K. (1999). Effects of nitric oxide inhibition on the spread of biotinylated dextran and on extracellular space parameters in the neostriatum of the male rat. Neuroscience.

[76] Chen K.C., Nicholson C. (2000). Changes in brain cell shape create residual extracellular space volume and explain tortuosity behaviour during osmotic challenge. Proc. Natl. Acad. Sci. USA.

[77] Yamaguchi Y. (2000). Lecticans: Organizers of the brain extracellular matrix. Cell. Mol. Life Sci..

[78] Fuxe K., Agnati L.F., Squire L.R. (2009). Cell–cell communication through the extracellular space. Encyclopedia of the Neuroscience.

[79] Marcoli M., Agnati L.F., Benedetti F., Genedani S., Guidolin D., Ferraro L., Maura G., Fuxe K. (2005). On the role of extracellular space on the holistic behavior of the brain. Rev. Neurosci..

[80] Nicholson C. (2001). Diffusion and related transport mechanisms in brain tissue. Rep. Prog. Phys. 64, 815–884; Syková, E. and Nicholson, C. Diffusion in brain extracellular space. Physiol. Rev..

[81] Odackal J., Colbourn R., Odackal N.J., Tao L., Nicholson C., Hrabetova S. (2017). Real-time Iontophoresis with Tetramethylammonium to Quantify Volume Fraction and Tortuosity of Brain Extracellular Space. J. Vis. Exp..

[82] Zhang H., Verkman A.S. (2010). Microfiberoptic measurement of extracellular space volume in brain and tumor slices based on fluorescent dye partitioning. Biophys. J..

[83] Dale H.H. (1935). Pharmacology and nerve-endings. Proc. R. Soc. Med..

[84] Eccles J.C. (1957). The Physiology of Nerve Cells.

[85] Hökfelt T. (1991). Neuropeptides in perspective: The last ten years. Neuron.

[86] Lundberg J.M. (1996). Pharmacology of cotransmission in the autonomic nervous system: Integrative aspects on amines, neuropeptides, adenosine triphosphate, amino acids and nitric oxide. Pharmacol. Rev..

[87] Hökfelt T., Broberger C., Xu Z.D., Sergeyev V., Ubink R., Diez M. (2000). Neuropeptides—An overview. Neuropharmacology.

[88] Hökfelt T., Arvidsson U., Cullheim S., Millhorn D., Nicholas A.P., Pieribone V., Seroogy K., Ulfhake B. (2000). Multiple messengers in descending serotonin neurons: Localization and functional implications. J. Chem. Neuroanat..

[89] Hökfelt T., Gutierrez R. (2009). Co-existence of neuromessenger molecules—A perspective, In Co-Existence and Co-Release of Classical Neurotransmitters.

[90] van den Pol A.N. (2012). Neuropeptide transmission in brain circuits. Neuron.

[91] Everitt B.J., Hökfelt T., Terenius L., Tatemoto K., Mutt V., Goldstein M. (1984). Differential co-existence of neuropeptide Y (NPY)-like immunoreactivity with catecholamines in the central nervous system of the rat. Neuroscience.

[92] Hökfelt T., Everitt B.J., Theodorsson-Norheim E., Goldstein M. (1984). Occurrence of neurotensin-like immunoreactivity in subpopulations of hypothalamic, mesencephalic, and medullary catecholamine neurons. J. Comp. Neurol..

[93] Johansson O., Hökfelt T., Pernow B., Jeffcoate S.L., White N., Steinbusch H.W., Verhofstad A.A., Emson P.C., Spindel E. (1981). Immunohistochemical support for three putative transmitters in one neuron: Coexistence of 5-hydroxytryptamine, substance P- and thyrotropin releasing hormone-like immunoreactivity in medullary neurons projecting to the spinal cord. Neuroscience.

[94] Glazer E.J., Steinbusch H., Verhofstad A., Basbaum A.I. (1981). Serotonin neurons in nucleus raphe dorsalis and paragigantocellularis of the cat contain enkephalin. J. Physiol..

[95] Vincent S.R., Satoh K., Armstrong D.M., Fibiger H.C. (1983). Substance P in the ascending cholinergic reticular system. Nature.

[96] Charnay Y., Léger L., Dray F., Bérod A., Jouvet M., Pujol J.F., Dubois P.M. (1982). Evidence for the presence of enkephalin in catecholaminergic neurones of cat locus coeruleus. Neurosci. Lett..

[97] Sperlágh B., Sershen H., Lajtha A., Vizi E.S. (1997). Co-release of endogenous ATP and [3H]noradrenaline from rat hypothalamic slices: Origin and modulation by α2-adrenoceptors. Neuroscience.

[98] Hökfelt T., Skirboll L., Rehfeld J.F., Goldstein M., Markey K., Dann O. (1980). A subpopulation of mesencephalic dopamine neurons projecting to limbic areas contains a cholecystokinin-like peptide: Evidence from immunohistochemistry combined with retrograde tracing. Neuroscience.

[99] Jo Y.-H., Role L.W. (2002). Cholinergic modulation of purinergic and GABAergic co-transmission at in vitro hypothalamic synapses. J. Neurophysiol..

[100] Oertel W.H., Graybiel A.M., Mugnaini E., Elde R.P., Schmechel D.E., Kopin I.J. (1983). Coexistence of glutamic acid decarboxylase- and somatostatin-like immunoreactivity in neurons of the feline nucleus reticularis thalami. J. Neurosci..

[101] Mori M., Heuss C., Gähwiler B.H., Gerber U. (2001). Fast synaptic transmission mediated by P2X receptors in CA3 pyramidal cells of rat hippocampal slice cultures. J. Physiol..

[102] Richardson P.J., Brown S.J. (1987). ATP release from affinity-purified rat cholinergic nerve terminals. J. Neurochem..

[103] Krügel U., Köles L., Illes P. (2013). Integration of neuronal and glial signalling by pyramidal cells of the rat prefrontal cortex; control of cognitive functions and addictive behaviour by purinergic mechanisms. Neuropsychopharmacol. Hung..

[104] Eckenstein F., Baughman R.W. (1984). Two types of cholinergic innervation in cortex, one co-localized with vasoactive intestinal polypeptide. Nature.

[105] Agnati L.F., Fuxe K., Torvinen M., Genedani S., Franco R., Watson S., Nussdorfer G.G., Leo G., Guidolin D. (2005). New methods to evaluate colocalization of fluorophores in immunocytochemical preparations as exemplified by a study on A2A and D2 receptors in chinese hamster ovary cells. J. Histochem. Cytochem..

[106] Bolte S., Cordelières F.P. (2006). A guided tour into subcellular colocalization analysis in light microscopy. J. Microsc..

[107] Dunn K.W., Kamocka M.M., McDonald J.H. (2011). A practical guide to evaluating colocalization in biological microscopy. Am. J. Physiol. Cell Physiol..

[108] Kupfermann I. (1991). Functional studies of cotransmission. Physiol. Rev..

[109] Cifuentes F., Morales M.A. (2021). Functional Implications of Neurotransmitter Segregation. Front. Neural Circuits.

[110] Svensson E., Apergis-Schoute J., Burnstock G., Nusbaum M.P., Parker D., Schiöth H.B. (2019). General principles of neuronal co-transmission: Insights from multiple model systems. Front. Neural Circuits.

[111] Marder E. (2012). Neuromodulation of neuronal circuits: Back to the future. Neuron.

[112] Brezina V. (2010). Beyond the wiring diagram: Signalling through complex neuromodulator networks. Philos. Trans. R. Soc. Lond. B Biol. Sci..

[113] Harris-Warrick R.M., Johnson B.R. (2010). Checks and balances in neuromodulation. Front. Behav. Neurosci..

[114] Nusbaum M.P., Blitz D.M., Marder E. (2017). Functional consequences of neuropeptide and small-molecule co-transmission. Nat. Rev. Neurosci..

[115] Granger A.J., Wallace M.L., Sabatini B.L. (2017). Multi-transmitter neurons in the mammalian central nervous system. Curr. Opin. Neurobiol..

[116] Kandel E., Koester J.D., Mack S.H., Siegelbaum S.A. (2021). Principles of Neuroscience.

[117] Linseman D.A., Benjamin C.W., Jones D.A. (1995). Convergence of angiotensin II and platelet-derived growth factor receptor signalling cascades in vascular smooth muscle cells. J. Biol. Chem..

[118] Guidolin D., Albertin G., Spinazzi R., Sorato E., Mascarin A., Cavallo D., Antonello M., Ribatti D. (2008). Adrenomedullin stimulates angiogenic response in cultured human vascular endothelial cells: Involvement of the vascular endothelial growth factor receptor 2. Peptides.

[119] Agnati L.F., Fuxe K., Zoli M., Rondanini C., Ogren S.O. (1982). New vistas on synaptic plasticity: The receptor mosaic hypothesis of the engram. Med. Biol..

[120] Fuxe K., Agnati L.F., Benfenati F., Celani M., Zini I., Zoli M., Mutt V. (1983). Evidence for the existence of receptor-receptor interactions in the central nervous system. Studies on the regulation of monoamine receptors by neuropeptides. J. Neural Transm..

[121] Kenakin T., Agnati L.F., Caron M., Fredholm B., Guidolin D., Kobilka B., Lefkowitz R.J., Lohse M., Woods A., Fuxe K. (2010). International workshop at the Nobel Forum, Karolinska Institutet on G protein-coupled receptors: Finding the words to describe monomers, oligomers, and their molecular mechanisms and defining their meaning. Can a consensus be reached?. J. Recept. Signal Transduct. Res..

[122] Marshall F.H., White J., Main M., Green A., Wise A. (1999). GABA(B) receptors function as heterodimers. Biochem. Soc. Trans..

[123] Fuxe K., Ferré S., Zoli M., Agnati L.F. (1998). Integrated events in central dopamine transmission as analyzed at multiple levels. Evidence for intramembrane adenosine A2A/dopamine D2 and adenosine A1/dopamine D1 receptor interactions in the basal ganglia. Brain Res. Rev..

[124] Bockaert J., Pin J.P. (1999). Molecular tinkering of G protein coupled receptors: An evolutionary success. EMBO J..

[125] Xie Z., Lee S.P., O’Dowd B.F., George S.R. (1999). Serotonin 5-HT1B and 5-HT1D receptors form homodimers when expressed alone and heterodimers when co-expressed. FEBS Lett..

[126] Franco R., Ferré S., Agnati L.F., Torvinen M., Ginés S., Hilion J., Casadò V., Lledò P., Zoli M., Lluis C. (2000). Evidence for adenosine/dopamine receptor interactions: Indications for heteromerization. Neuropsychopharmacology.

[127] Lee S.P., Xie Z., Varghese G., Nguyen T., O’Dowd B.F., George S.R. (2000). Oligomerization of dopamine and serotonin receptors. Neuropsychopharmacology.

[128] Overton M.C., Blumer K.J. (2000). G protein-coupled receptors function as oligomers in vivo. Curr. Biol..

[129] Zeng F., Wess J. (2000). Molecular aspects of muscarinic receptor dimerization. Neuropsychopharmacology.

[130] Angers S., Salahpour A., Bouvier M. (2001). Biochemical and biophysical demonstration of GPCR oligomerization in mammalian cells. Life Sci..

[131] Dean M.K., Higgs C., Smith R.E., Bywater R.P., Snell C.R., Scott P.D., Upton G.J., Howe T.J., Reynolds C.A. (2001). Dimerization of G protein-coupled receptors. J. Med. Chem..

[132] Kenakin T. (2002). Drug efficacy at G protein-coupled receptors. Annu. Rev. Pharmacol. Toxicol..

[133] Waldhoer M., Fong J., Jones R.M., Lunzer M.M., Sharma S.K., Kostenis E., Portoghese P.S., Whistler J.L. (2005). A heterodimer-selective agonist shows *in vivo* relevance of G protein-coupled receptor dimers. Proc. Natl. Acad. Sci. USA.

[134] Changeaux J.P., Christopoulos A. (2017). Allosteric modulation as a unifying mechanism for receptor function and regulation. Diabetes Obes. Metab..

[135] Guidolin D., Agnati L.F., Marcoli M., Borroto-Escuela D., Fuxe K. (2015). G-protein-coupled receptor type A heteromers as an emerging therapeutic target. Expert Opin. Ther. Targets.

[136] Farran B. (2017). An update on the physiological and therapeutic relevance of GPCR oligomers. Pharmacol. Res..

[137] Guidolin D., Marcoli M., Tortorella C., Maura G., Agnati L.F. (2018). G protein-coupled receptor-receptor interactions give integrative dynamics to intercellular communication. Rev. Neurosci..

[138] Gainetdinov R.R., Premont R.T., Bohn L.M., Lefkowitz R.J., Caron M.G. (2004). Desensitization of G protein-coupled receptors and neural functions. Annu. Rev. Neurosci..

[139] Smith J.S., Rajagopal S. (2016). The β-arrestin: Multifunctional regulators of G protein-coupled receptors. J. Biol. Chem..

[140] Guidolin D., Marcoli M., Tortorella C., Maura G., Agnati L.F. (2019). Receptor-receptor interactions as a widespread phenomenon: Novel targets for drug development?. Front. Endocrinol..

[141] Marullo S., Bouvier M. (2007). Resonance energy transfer approaches in molecular pharmacology and beyond. Trends Pharmacol. Sci..

[142] Herrick-Davis K., Grinde E., Cowan A., Mazurkiewicz J.E. (2013). Fluorescence correlation spectroscopy analysis of serotonin, adrenergic, muscarinic, and dopamine receptor dimerization: The oligomer number puzzle. Mol. Pharmacol..

[143] Trifilieff P., Rives M.L., Urizar E., Piskorowski R.A., Vishwasrao H.D., Castrillon J., Schmauss C., Slättman M., Gullberg M., Javitch J.A. (2011). Detection of antigen interactions ex vivo by proximity ligation assay: Endogenous dopamine D2-adenosine A2A receptor complexes in the striatum. Biotechniques.

[144] Borroto-Escuela D.O., Romero-Fernandez W., Garriga P., Ciruela F., Narvaez M., Tarakanov A.O., Palkovits M., Agnati L.F., Fuxe K. (2013). G protein-coupled receptor heterodimerization in the brain. Methods Enzymol..

[145] Ullman E.F., Kirakossian H., Switchenko A.C., Ishkanian J., Ericson M., Wartchow C.A., Pirio M., Pease J., Irvin B.R., Singh S. (1996). Luminescent oxygen channeling assay (LOCI): Sensitive, broadly applicable homogeneous immunoassay method. Clin Chem..

[146] Fernández-Dueñas V., Gòmez-Soler M., Valle-Leòn M., Watanabe M., Ferrer I., Ciruela F. (2019). Revealing adenosine A2A-dopamine D2 receptor heteromers in Parkinson’s disease post-mortem brain through a new AlphaScreen-based approach. Int. J. Mol. Sci..

[147] Pelassa S., Guidolin D., Venturini A., Averna M., Frumento G., Campanini L., Bernardi R., Cortelli P., Calandra Buonaura G., Maura G. (2019). A2A-D2 heteromer on striatal astrocytes: Biochemical and biophysical evidence. Int. J. Mol. Sci..

[148] Guidolin D., Tortorella C., Marcoli M., Maura G., Agnati L.F. (2021). Receptor-receptor interactions and glial cell functions with a special focus on G protein-coupled receptors. Int. J. Mol. Sci..

[149] Di Liberto V., Borroto-Escuela D.O., Frinchi M., Verdi V., Fuxe K., Belluardo N., Mudò G. (2017). Evidence of muscarinic acetylcholine receptor (mAChR) and fibroblast growth factor receptor (FGFR) heteroreceptor complexes and their enhancement of neurite outgrowth in neural hippocampal cultures. Biochim. Biophys. Acta.

[150] Agnati L.F., Guidolin D., Vilardaga J.P., Ciruela F., Fuxe K. (2010). On the expanding terminology in the GPCR field: The meaning of receptor mosaics and receptor heteromers. J. Receptor Signal Transduct. Res..

[151] Alemany R., Perona J.S., Sánchez-Dominguez J.M., Montero E., Cañizares J., Bressani R., Escribà P.V., Ruiz-Gutierrez V. (2007). G protein-coupled receptor systems and their lipid environment in health disorders during aging. Biochim. Biophys. Acta.

[152] Bockaert J., Perroy J., Becamel C., Marin P., Fagni L. (2010). GPCR interacting proteins (GIPs) in the nervous system: Roles in physiology and pathologies. Annu. Rev. Pharmacol. Toxicol..

[153] Franco R., Ciruela F., Casado V., Cortes A., Canela E.I., Mallol J., Agnati L.F., Ferré S., Fuxe K., Lluis C. (2005). Partners for adenosine A1 receptors. J. Mol. Neurosci..

[154] Ciruela F., Canela L., Burgueno J., Soriguera A., Cabello N., Canela E.I., Casado V., Cortes A., Mallol J., Woods A.S. (2005). Heptaspanning membrane receptors and cytoskeletal/scaffolding proteins: Focus on adenosine, dopamine, and metabotropic glutamate receptor function. J. Mol. Neurosci..

[155] Fuxe K., Borroto-Escuela D.O., Ciruela F., Guidolin D., Agnati L.F. (2014). Receptor-receptor interactions in heteroreceptor complexes: A new principle in biology. Focus on their role in learning and memory. Neurosci. Discov..

[156] Simons M., Raposo G. (2009). Exosomes—Vesicular carriers for intercellular communication. Curr. Opin. Cell Biol..

[157] Lakkaraju A., Rodriguez-Boulan E. (2008). Itinerant exosomes: Emerging roles in cell and tissue polarity. Trends Cell Biol..

[158] Smalheiser N.R. (2007). Exosomal transfer of proteins and RNAs at synapses in the nervous system. Biol. Direct..

[159] Calzolari A., Raggi C., Deaglio S., Sposi N.M., Stafsnes M., Fecchi K., Parolini I., Malavasi F., Peschle C., Sargiacomo M. (2006). TfR2 localizes in lipid raft domains and is released in exosomes to activate signal transduction along the MAPK pathway. J. Cell Sci..

[160] Guescini M., Leo G., Genedani S., Carone C., Pederzoli F., Ciruela F., Guidolin D., Stocchi V., Mantuano M., Borroto-Escuela D.O. (2012). Microvesicle and tunneling nanotube mediated intercellular transfer of g-protein coupled receptors in cell cultures. Exp. Cell Res..

[161] Woods A., Genedani S., Carone C., Guescini M., Leo G., Stocchi V., Borroto-Escuela D., Morales M., Guidolin D., Fuxe K. (2013). Can cocaine by affecting A2a-D2 colocalisation in lipid rafts modify the intercellular transfer of A2a and D2 via the roamer type of volume transmission?. J. Extracell. Ves..

[162] Venturini A., Passalacqua M., Pelassa S., Pastorino F., Tedesco M., Cortese K., Gagliani M.C., Leo G., Maura G., Guidolin D. (2019). Exosomes from astrocyte processes: Signaling to neurons. Front. Pharmacol..

[163] Webster R. (2001). Neurotransmitters, Drugs and Brain Function.

[164] Pich E.M., Benfenati F., Farabegoli C., Fuxe K., Meller E., Aronsson M., Goldstein M., Agnati L.F. (1987). Chronic haloperidol affects striatal D2-dopamine receptor reappearance after irreversible receptor blockade. Brain Res..

[165] Carta M., Carlsson T., Kirik D., Björklund A. (2007). Dopamine released from 5-HT terminals is the cause of L-DOPA-induced dyskinesia in parkinsonian rats. Brain.

[166] Malave L., Zuelke D.R., Uribe-Cano S., Starikov L., Rebholz H., Friedman E., Qin C., Li Q., Bezard E., Kottmann A.H. (2021). Dopaminergic co-transmission with sonic hedgehog inhibits abnormal involuntary movements in models of Parkinson’s disease and L-Dopa induced dyskinesia. Commun. Biol..

[167] Gariano R.F., Groves P.M. (1989). A mechanism for the involvement of colocalized neuropeptides in the actions of antipsychotic drugs. Biol. Psychiatry.

[168] Zoli M., Jansson A., Syková E., Agnati L.F., Fuxe K. (1999). Volume transmission in the CNS and its relevance for neuropsychopharmacology. Trends Pharmacol. Sci..

[169] Bortolanza M., Padovan-Neto F.E., Cavalcanti-Kiwiatoski R., Dos Santos-Pereira M., Mitkowski M., Raisman-Vozari R., Del-Bel E. (2015). Are cyclooxygenase-2 and nitric oxide involved in the dyskinesia of Parkinson’s disease induced by L-DOPA?. Philos. Trans. R. Soc. Lond. B Biol. Sci..

[170] Liu C.M., Spaulding M.O., Rea J.J., Noble E.E., Kanoski S.E. (2021). Oxytocin and food intake control: Neural, behavioral and signaling mechanisms. Int. J. Mol. Sci..

[171] Morganstern I., Gulati G., Leibowitz S.F. (2020). Role of melanin-concentrating hormone in drug use disorders. Brain Res..

[172] Alpár A., Zahola P., Hanics J., Hevesi Z., Korchynska S., Benevento M., Pifl C., Zachar G., Perugini J., Severi I. (2018). Hypothalamic CNTF volume transmission shapes cortical noradrenergic excitability upon acute stress. EMBO J..

[173] Fuxe K., Borroto-Escuela D.O., Guidolin D., Tarakanov A., Agnati L.F. (2014). The balance and integration of different forms of volume and wiring transmission in the CNS. Relevance for schizophrenia. Neurol. Psychiatry Brain Res..

[174] Bechter K. (2013). Updating the mild encephalitis hypothesis of schizophrenia. Prog. Neuropsychopharmacol. Biol. Psychiatry.

[175] Borroto-Escuela D.O., Perez De La Mora M., Manger P., Narváez M., Beggiato S., Crespo-Ramírez M., Navarro G., Wydra K., Díaz-Cabiale Z., Rivera A. (2018). Brain dopamine transmission in health and Parkinson’s disease: Modulation of synaptic transmission and plasticity through volume transmission and dopamine heteroreceptors. Front. Synaptic Neurosci..

[176] Vizi E.S., Fekete A., Karoly R., Mike A. (2010). Non-synaptic receptors and transporters involved in brain functions and targets of drug treatment. Br. J. Pharmacol..

[177] Fuxe (2015). K.; Guidolin, D.; Agnati, L.F.; Borroto-Escuela, D.O. Dopamine heteroceptor complexes as therapeutic targets in Parkinson’s disease. Expert Opin. Ther. Targets.

[178] Sahlholm K., Gómez-Soler M., Valle-León M., Lopez-Cano M., Taura J.J., Ciruela F., Fernandez-Dueñas V. (2017). Antipsychotic-like efficacy of dopamine D2 receptor-biased ligands is dependent on adenosine A2A receptor expression. Mol. Neurobiol..

[179] Bushlin I., Gupta A., Stockton S.D., Miller L.K., Devi L.A. (2012). Dimerization with cannabinoid receptors allosterically modulates δ opioid receptor activity during neuropathic pain. PLoS ONE.

[180] Gomes I., Fujita W., Chandrakala M.V., Devi L.A. (2013). Disease-specific heteromerization of G-protein-coupled receptors that target drugs of abuse. Prog. Mol. Biol. Transl. Sci..

[181] Kern A., Albarran-Zeckler R., Walsh H.E., Smith R.G. (2012). Apo-ghrelin receptor forms heteromers with DRD2 in hypothalamic neurons and is essential for anorexigenic effects of DRD2 agonism. Neuron.

[182] Le Naour M., Lunzer M.M., Powers M.D., Kalyuzhny A.E., Benneyworth M.A., Thomas M.J., Portoghese P.S. (2014). Putative opioid heteromers as targets for developing analgesics free of adverse effects. J. Med. Chem..

[183] Guidolin D., Marcoli M., Tortorella C., Maura G., Agnati L.F. (2020). Adenosine A_2A_-dopamine D_2_ receptor-receptor interactions in neurons and astrocytes: Evidence and perspectives. Prog. Mol. Biol. Transl. Sci..

[184] Ferré S., Fredholm B.B., Morelli M., Popoli P., Fuxe K. (1997). Adenosine-dopamine receptor-receptor interactions as an integrative mechanism in the basal ganglia. Trends Neurosci..

[185] Canals M., Marcellino D., Fanelli F., Ciruela F., De Benedetti P., Goldberg S.R., Neve K., Fuxe K., Agnati L.F., Woods A.S. (2003). Adenosine A2A-dopamine D2 receptor-receptor heteromerization: Qualitative and quantitative assessment by fluorescence and bioluminescence energy transfer. J. Biol. Chem..

[186] Agnati L.F., Guidolin D., Leo G., Carone C., Genedani S., Fuxe K. (2010). 2010. Receptor-receptor interactions: A novel concept in brain integration. Prog. Neurobiol..

[187] Díaz-Cabiale Z., Hurd Y., Guidolin D., Finnman U.B., Zoli M., Agnati L.F., Vanderhaeghen J.J., Fuxe K., Ferré S. (2001). Adenosine A2A agonist CGS 21680 decreases the affinity of dopamine D2 receptors for dopamine in human striatum. Neuroreport.

[188] Fuxe K., Marcellino D., Borroto-Escuela D.O., Guescini M., Fernandez-Duenas V., Tanganelli S., Rivera A., Ciruela F., Agnati L.F. (2010). Adenosine-dopamine interactions in the pathophysiology and treatment of CNS disorders. CNS Neurosci. Ther..

[189] Xu K., Bastia E., Schwarzschild M. (2005). Therapeutic potential of adenosine A2A receptor antagonists in Parkinson’s disease. Pharmacol. Ther..

[190] U.S. Food&Drug Administration Drugs@FDA: FDA Approved Drug Products. NDA 022075. https://www.accessdata.fda.gov/scripts/cder/daf/index.cfm?event=overview.process&varApplNo=022075.

[191] Cervetto C., Venturini A., Guidolin D., Maura G., Passalacqua M., Tacchetti C., Cortelli P., Genedani S., Candiani S., Ramoino P. (2018). Homocysteine and A2A-D2 Receptor-Receptor Interaction at Striatal Astrocyte Processes. J. Mol. Neurosci..

[192] Daniels D.J., Lenard N.R., Etienne C.L., Law P.-Y., Roerig S.C., Portoghese P.S. (2005). Opioid-induced tolerance and dependence in mice is modulated by the distance between pharmacophores in a bivalent ligand series. Proc. Natl. Acad. Sci. USA.

[193] Harada K., Kamiya T., Tsuboi T. (2015). Gliotransmitter release from astrocytes: Functional, developmental, and pathological implications in the brain. Front. Neurosci..

[194] Gourine A.V., Kasymov V., Marina N., Tang F., Figueiredo M.F., Lane S., Teschemacher A.G., Spyer K.M., Deisseroth K., Kasparov S. (2010). Astrocytes control breathing through pH-Dependent release of ATP. Science.

[195] Yang Y., Ge W., Chen Y., Zhang Z., Shen W., Wu C., Poo M., Duan S. (2003). Contribution of astrocytes to hippocampal long-term potentiation through release of D-serine. Proc. Natl. Acad. Sci. USA.

[196] Reichenbach A., Derouiche A., Kirchhoff F. (2010). Morphology and dynamics of perisynaptic glia. Brain Res. Rev..

[197] Basilico B., Ferrucci L., Ratano P., Golia M.T., Grimaldi A., Rosito M., Ferretti V., Reverte I., Sanchini C., Marrone M.C. (2022). Microglia control glutamatergic synapses in the adult mouse hippocampus. Glia.

[198] Schiera G., Di Liegro C.M., Di Liegro I. (2019). Cell-to-cell communication in learning and memory: From neuro- and glio-transmission to information exchange mediated by extracellular vesicles. Int. J. Mol. Sci..

[199] Franco R., Reyes-Resina I., Aguinaga D., Lillo A., Jiménez J., Raïch I., Borroto-Escuela D.O., Ferreiro-Vera C., Canela E.I., De Medina V.S. (2019). Potentiation of cannabinoid signaling in microglia by adenosine A2A receptor antagonists. Glia.

[200] Hernandez-Sosa A., Fisahn A., Fuxe K., Borroto-Escuela D.O. (2020). Existence of FGFR1-5-HT1AR heteroreceptor complexes in hippocampal astrocytes. Putative link to 5-HT and FGF2 modulation of hippocampal gamma oscillations. Neuropharmacology.

[201] Bullmore E., Sporns O. (2012). The economy of brain network organization. Nat. Rev. Neurosci..

[202] Agnati L.F., Baluška F., Barlow P.W., Guidolin D. (2009). Mosaic, self-similarity logic, and biological attraction principles: Three explanatory instruments in biology. Commun. Integr. Biol..

[203] Krishnan V., Nestler E.J. (2008). The molecular neurobiology of depression. Nature.

[204] Finnerup N.B., Kuner R., Jensen T.S. (2021). Neuropathic pain: From mechanisms to treatment. Physiol. Rev..

[205] Kabanova A., Pabst M., Lorkowski M., Braganza O., Boehlen A., Nikbakht N., Pothmann L., Vaswani A.R., Musgrove R., Di Monte D.A. (2015). Function and developmental origin of a mesocortical inhibitory circuit. Nat. Neurosci..

[206] Pérez-López J.L., Contreras-López R., Ramírez-Jarquín J.O., Tecuapetla F. (2018). Direct glutamatergic signaling from midbrain dopaminergic neurons onto pyramidal prefrontal cortex neurons. Front. Neural Circuits.

[207] Borroto-Escuela D.O., Brito I., Romero-Fernandez W., Di Palma M., Oflijan J., Skieterska K., Duchou J., Van Craenenbroeck K., Suárez-Boomgaard D., Rivera A. (2014). The G protein-coupled receptor heterodimer network (GPCR-HetNet) and its hub components. Int. J. Mol. Sci..

[208] Gamo N.J., Lur G., Higley M.J., Wang M., Paspalas C.D., Vijayraghavan S., Yang Y., Ramos B.P., Peng K., Kata A. (2015). Stress impairs prefrontal cortical function via D1 dopamine receptor interactions with HCN channels. Biol. Psychiatry.

[209] Gurevich V.V., Gurevich E.V. (2008). How and why do GPCRs dimerize?. Trends Pharmacol. Sci..

[210] Balakrishnan A., Hemmen K., Choudhuri S., Krohn J.H., Jansen K., Friedrich M., Beliu G., Sauer M., Lohse M.J., Heinze K.G. (2022). Unraveling the hidden temporal range of fast β_2_-adrenergic receptor mobility by time-resolved fluorescence. Commun. Biol..

[211] Hoestgaard-Jensen K., O’Connor R.M., Dalby N.O., Simonsen C., Finger B.C., Golubeva A., Hammer H., Bergmann M.L., Kristiansen U., Krogsgaard-Larsen P. (2013). The ortosteric GABAA receptor liganafridid Thio-4-PIOL displays distinctly different functional properties at synaptic and extrasynaptic receptors. Br. J. Pharmacol..

[212] Middendorp S.J., Maldifassi M.C., Baur R., Sigel E. (2015). Positive modulation of synaptic and extrasynaptic GABAA receptors by an antagonist of the high affinity benzodiazepine binding site. Neuropharmacology.

[213] Afridi R., Kim J.-H., Rahman M.H., Suk K. (2020). Metabolic regulation of glial phenotypes: Implications in neuron-glia interactions and neurological disorders. Front. Cell Neurosci..

